# Treatment of Typhoid Fever

**Published:** 1898-07

**Authors:** 


**Treatment of Typhoid Fever.**—So many inquiries have been received by us (*Memphis Med. Jour.*) and the Sisters of St. Joseph's Hospital, Memphis, relative to the treatment of typhoid fever so successfully used by the former house-physician of that institution, Dr. Alfred Moore, that we produce the prescription from the March issue of the *Monthly:*

℞ Salol, ................................ʒ j.

Thymol, ....................gr. xxxvi.

Bismuth subnitrat,...............ʒ ij.

Muc. acaciæ, .......................℥ ij.

Syr. tolutan,.......................℥ iv.

M. Sig: Tablespoonful three times daily.—*Medical Review of Reviews.*

